# MS for investigation of time-dependent protein adsorption on surfaces in complex biological samples

**DOI:** 10.4155/fso.15.32

**Published:** 2015-11-01

**Authors:** Torgny Undin, Sara Bergström Lind, Andreas P Dahlin

**Affiliations:** 1 Department of Chemistry-BMC, Science for Life Laboratory, Uppsala University, PO Box 599, SE-751 24 Uppsala, Sweden; 2 Department of Engineering Sciences, Uppsala University, PO Box 534, SE-751 21 Uppsala, Sweden

**Keywords:** MS, on-surface digestion, polycarbonate membrane, time-resolved protein adsorption, ventricular cerebrospinal fluid

## Abstract

**Aim:**

This study aims at developing a nondestructive way for investigating protein adsorption on surfaces such as biomaterials using mass spectrometry.

**Methods:**

Ventricular cerebrospinal fluid in contact with poly carbonate membranes were used as adsorption templates and on-surface enzymatic digestion was applied to desorb proteins and cleave them into peptides. Mass spectrometric analysis provided both protein identification and determination of protein specific adsorption behavior.

**Results:**

In general, the adsorption increased with incubation time but also protein-specific time-resolved adsorption patterns from the complex protein solution were discovered.

**Conclusion:**

The method developed is a promising tool for the characterization of biofouling, which sometimes causes rejection and encapsulation of implants and can be used as complement to other surface analytical techniques.

## Abstract (lay abstract)

One problem associated with artificial materials in the body is that proteins in the body interact with the surface, which sometimes causes rejection of the implant. In this study, a method for investigating the time-dependent protein adsorption on a surface originating from a complex biological protein solution was developed. Compared with other surface analyses, this method can identify what proteins that adsorbs on the surface. In addition, determination of protein-specific adsorption behavior in relation to incubation was possible. Generally, more proteins adsorbed to the surface with increase in incubation time.

**Figure 1.**
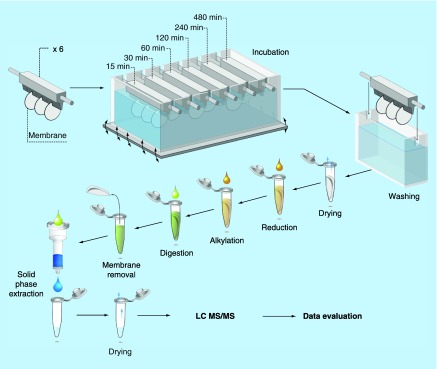
**Representation of the experimental sampling set up.** Two of the membranes were used for MS measurements for each time point.

**Figure 2.**
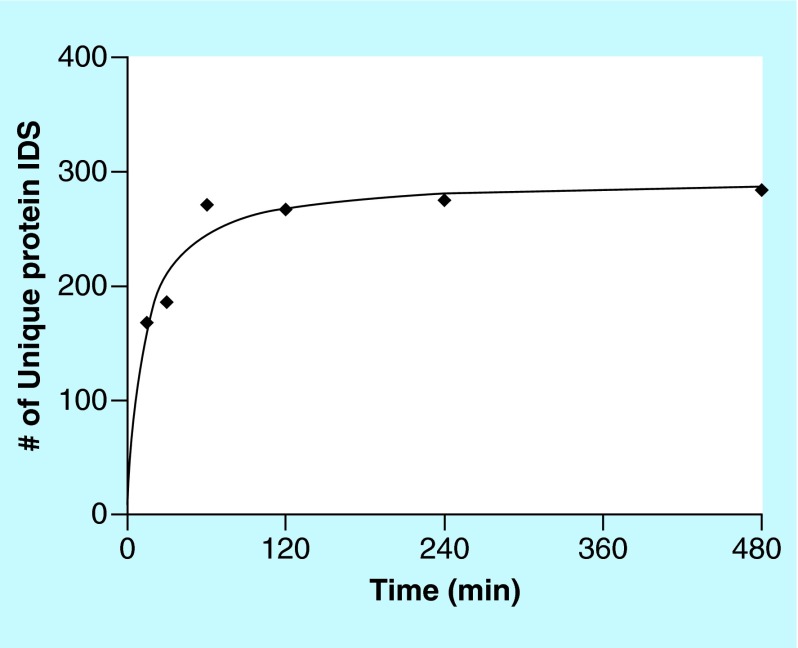
**Number of adsorbed proteins as function of time.** The total number of adsorbed proteins at each time point, detected with at least two unique peptides, plotted against time of adsorption. Two experimental replicates were used at every time point.

**Figure 3.**
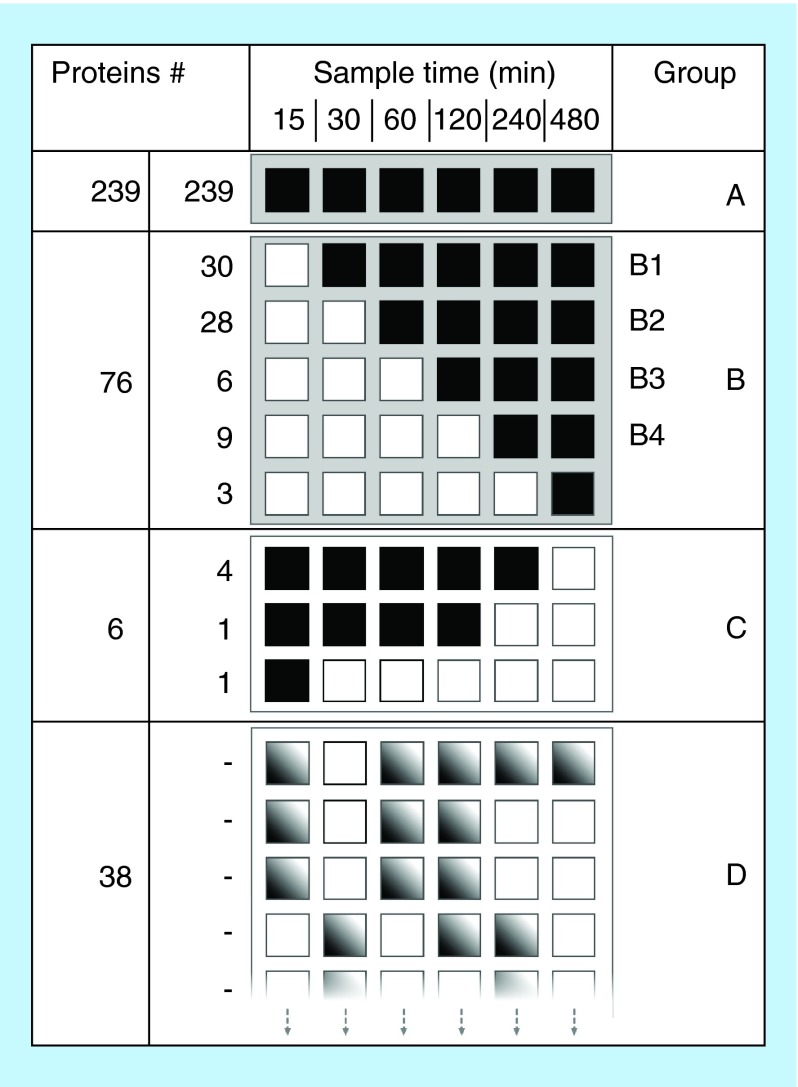
**Protein adsorption behavior grouping.** Counted and grouped proteins according to adsorption time for detection (black square). The adsorption time is presented on the x-axis and group name and number of proteins detected per group are presented on the y-axis. Group A are proteins that are adsorbed at every time point. Group B has an initial first adsorption delay and thereafter detected at following time points. Group B is further divided into sub groups depending on when first adsorption is detected. Group C are proteins that are adsorbed from the first time point to a later point. Group D are proteins that show no clear adsorption pattern indicated by nonuniform gray squares illustrating randomness. Proteins were identified with at least two unique peptides at one time point in the time interval.

**Figure 4:**
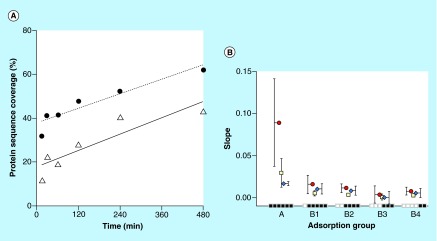
**Evaluation of protein adsorption behaviour.** **(A)** Protein sequence coverage change. The slope of protein sequence change depending on time for serum albumin (solid line) and cystatin-C (dashed line) is the linear regression of all sample points (15–480 min). Proteins identified with two unique peptides at least at one time point in interval. **(B)** Mean slope parameter values. The mean slope value for protein score (red circle), peptide spectrum match (white square) and protein sequence coverage (blue diamond), for different adsorbing behavior groups, which from left to right are: A (15–480), B_1_ (30–480), B_2_ (60–480), B_3_ (120–480), B_4_ (240–480) min. Proteins identified with two unique peptides at least at one time point in the time interval.

**Figure 5.**
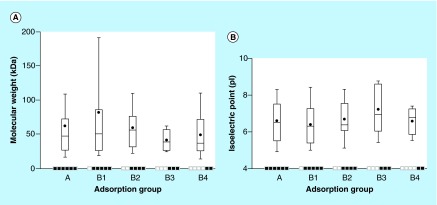
**Protein weight and isoelectric point comparison with adsorption behavior.** Median (straight bar in box), mean (black spot) and 90% interval of the molecular weight (y-axis in **[A]**) and isoelectric point (y-axis in **[B]**) for the different proteins adsorbed at different incubation times and detected on all the later time points (x-axis). From left to right: A (15–480), B_1_ (30–480), B_2_ (60–480), B_3_ (120–480), B_4_ (240–480) min. Proteins identified with two unique peptides at least at one time point in the time interval.

Materials that are brought in contact with biological matrices *in vivo*, for example, blood, plasma, serum, cerebrospinal fluid (CSF), are often referred to as biomaterials. The immediate response, when a foreign material is implanted, is the spontaneous formation of a protein layer on the inserted material [1]. Protein adsorption initiates cascade processes that lead to biofouling [1] and finally formation of a tissue scar, that is, encapsulation [2,3].

The complex and dynamic adsorption mechanism is, despite years of intense research, not fully explained since it depends on many parameters. These include, first, the physical and chemical properties of the solid material such as its hydrophobicity, charge and roughness. Second, parameters as the composition, complexity and concentration of the protein sample, all influence the adsorption mechanism together with the chemical properties of the liquid in which the proteins are present for example, pH and ion strength. Lastly, the physical properties of the complete system, which include sample temperature and different convective fluxes [4] need to be monitored and controlled.

Some of the fundamental issues regarding the mechanisms behind protein adsorption are; the reversibility/irreversibility of protein adsorption, the so-called Vroman effect [5], capacity of multilayer adsorptions [6], energetics [7] and applicability of thermodynamic/computational models [8].

There are many ways to analyze protein adsorption, including radiolabeling, immunoassays, spectroscopy, quartz crystal microbalance (QCM), atomic force microscopy (AFM), surface plasmon resonance (SPR), Fourier transform infrared spectroscopy (FTIR), ellipsometry and MS [9]. Except for MS, the methods are not capable of providing protein identification without any *a priori* information. It is therefore difficult or impossible to discriminate between different adsorbed proteins originating from complex biological samples. MS-based methods are selective and sensitive and well suited for peptide and protein determination in complex biological samples. They can readily be used to simultaneously identify and quantify a large number of proteins. Additionally, MS can also be used in targeted mode to specifically monitor a protein of interest. For some applications, MS analysis can be performed directly on some material surfaces that contain the adsorbed proteins using time-of-flight secondary ion MS (ToF-SIMS) [10,11], but lack in protein identification compared with MS systems is capable of tandem MS fragmentation. For the use of more common MS analyses, it is necessary to desorb the proteins from the surface to solution, often by the use of detergents such as sodium dodecylsulphate [12,13] and salt. Detergents and salts are incompatible with direct MS detect, wherefore further sample preparation steps are necessary. However, this in turn can impair protein detection.

Recently [14,15] our group used a slight modified method for analyzing adsorbed proteins on arbitrary surfaces. By using an on-surface enzymatic digestion (oSED) protocol, the proteins were reduced, alkylated and enzymatically cleaved without excessive protein desorption from the membrane surfaces. The oSED approach has also been demonstrated on fragile hollow-fiber membranes used for microdialysis sampling and fmol amounts of proteins could be detected using matrix-assisted laser desorption/ionization MS [16]. In 1987, the first use of trypsin onto adsorbed proteins was reported when producing pure peptides from low amounts of N-terminally blocked proteins electroblotted onto nitrocellulose after polyamide gel electrophoresis [17]. A number of studies have used similar principles, for example, with the identification of several proteins in the coronal sections of a rat brain [18], on reversed-phase stationary phases to detect protein interaction sites [19], and also for on-bead enzymatic digestion using trypsin in an integrated MALDI target for antibody affinity capture [20], to mention a few.

In the current study, we have developed a method based on the oSED approach coupled to ESI-tandem MS for measurement of time-dependent protein adsorption from a complex ventricular CSF (vCSF) sample on a model surface made of polycarbonate (PC). PC was chosen as material due to having intermediate hydrophobic properties (contact angle ˜80°) and its use in some membranes for microdialysis, which also can suffer from protein adsorption. We have qualitatively investigated how complex protein adsorption changed over a period of 480 min using data easy to identify even for the non-MS-specialized reader.

## Experimental

### Chemicals & reagents

ACN and acetic acid (HAc) were obtained from Merck (Darmstadt, Germany). IAA, urea, NH4HCO3, DTT and TFA were obtained from Sigma-Aldrich (MO, USA). Trypsin was of sequence-grade from Roche Diagnostics (Basel, Switzerland). Ultrapure water was produced by a Milli-Q+ system, Millipore Corp. (MA, USA). Electron track etched polycarbonate filter membranes with a diameter of 13 mm and a pore size of 0.22 μm was obtained from Millipore SAS (Molsheim, Alsace, France) and used as a model surfaces for protein adsorption.

### Cerebrospinal fluid collection

CSF was collected by ventricular drainage from neurointensive care patients with invasive intracranial pressure monitoring owing to severe spontaneous subarachnoid hemorrhage. The total protein amount in the pooled vCSF was measured to 78 ± 5 mg/l (n = 3) using Bradford Coomassie^®^ Brilliant Blue G-250 protein assay (595 nm, Bovine Serum Albumin as standard, Bio-Rad, CA, USA). The vCSF was centrifuged before being stored in a -70°C freezer. In total, 400 ml vCSF was collected, pooled and finally divided in 10 aliquots, each containing 40 ml vCSF. The aliquots were stored in -80°C until further use. Permission to use ventricular CSF has been granted by the Regional Ethics Committee (Dnr UPS 01-367).

### Protein adsorption in vCSF

Six in-house made polydimethylsiloxane holders were used to hold three membranes each, of which two were used for MS experiments. The holders were further placed into a beaker according to Figure 1. The distance between each membrane was between 5 mm and 10 mm, due to different curving of the membrane. The holders were immersed into a beaker containing 200 ml pooled vCSF.

The beaker was placed in a heating block at 37°C, and a rocker kept the vCSF under gentle constant motion over the membrane surfaces. After each incubation time, the membranes were rinsed in water at 37°C for 5 min. The excess rinsing water was removed using a blotting paper on the edge of the membrane before transferring the membrane into a vial where it was completely dried using a gentle flow of nitrogen gas.

### On-surface enzymatic digestion

The dried membranes in the vials were cut into four pieces equal in size in order to be able to be totally soaked by added solutions. First, a 4 mM DTT solution based on 200 μl 50 mM ammonium bicarbonate buffer and 20 μl 45 mM DDT solution was added to reduce protein disulfide bonds. Then, the vials were placed on a heater block for 15 min at 50°C. After reduction, the vial was cooled down to 22°C before an addition of 20 μl 100 mM IAA to a concentration of 80 mM. The reaction was performed in room temperature (˜22°C) and darkness for 15 min. After the addition of 1 μg trypsin to every membrane and incubation in 37°C for 24 h on the rocker, the membranes were removed and the peptide solution was dried down in a SpeedVac (Thermo Fisher Scientific, Inc., MA, USA). Prior to the MS analysis in the different MS systems, the samples were desalted, using C18 SPE columns according to previously described procedure [15]. Finally, the dried peptide solution was resuspended in 0.1% FA solution.

### Column packing

The columns were in-house packed 15 cm fused silica columns with integrated emitter (75 μm i.d., 375 μm o.d.). The columns were packed with a methanol slurry of reversed phase, fully end-capped Reprosil-Pur C18-AQ 3 μm resin with a pore diameter of 120 Å (Dr. Maisch GmbH, Ammerbuch-Entringen, Germany) with the help of a pressure packing device operated at 50 bars.

### Systems for protein identification

Two LC–MS systems were used: The FTICR-system included an online Agilent 1100 converted nano-LC (Agilent Technologies, CA, USA) system connected to a 7 T hybrid linear in trap Fourier transform Ultra MS (Thermo). The Orbitrap system had an online nano-LC (EASY-nLC II, Thermo) system connected to a linear in trap Orbitrap Velos Pro (Thermo).

The sample injection volumes were 4 μL. During analysis, the samples were kept at 10°C. Gradient elution was utilized with solvent A: water/ACN/FA: 98/2/0.1 v/v% and solvent B: water/ACN/FA: 2/98/0.1 v/v% (FTICR system used HAc instead of FA in both buffers) at a flow rate of 200 nL/min. The elution gradient (% solvent B in A) was: 4–35% over 70 min followed by 35–50% over 18 min.

The source parameters were: ion spray voltage, 1.7 and 2.1 kV, for FT-ICR and Orbitrap, respectively. Interface heater temperature was 240°C for both systems. Precursors were selected by using a survey scan of resolution 100,000 at FWHM, m/z 400–1500 for FT-ICR and 400–2000 for the Orbitrap, both instrument used the charges of +2, +3 and +4. Product ion scans (MS/MS) fragmentation using CID of the five most intense precursor ions on the FT-ICR, and ten most intense for the Orbitrap, were then performed. Precursor ions selected for product ion scans were excluded for fragmentation for the following 150 s on the FT-ICR and 30 s on the Orbitrap. For both systems, the Xcalibur (Thermo Scientific, version 2.0.7 for FT-ICR and 2.1.0 for the Orbitrap) software was used for data acquisition.

### Data evaluation

Data were processed using commercially available softwares. The Protein Discoverer (Thermo Scientific, v. 1.4.0.288) software was used with the embedded SEQUEST [21] database search program for an automatic protein identification and ratio determination against the Uniprot/SwissProt database for *Homo sapien*s (release: 2013-02-13). The search parameters were set to: Digestion enzyme: Trypsin; Max missed cleavages: 2; Highest charge state: 4; Dynamic modification: Oxidation(M), Deamination(N, Q); Static modification: Carbamidomethylation (C); Precursor mass tolerance: 0.02 Da; Fragment mass tolerance: 0.7 Da.

The criteria of protein identification were based on at least two unique (not present in other proteins) and significant (q-value < 0.05) peptides per protein. Peptides were scored in Proteome Discoverer using the embedded Percolator function (version 2.04), and selected if the false-discovery rate (FDR) level was lower than 0.05.

## Result & discussion

The adsorption of proteins in body fluids and tissue to material surfaces of, for example, sampling devices is not fully understood and there is a need for novel methods that can describe the adsorption of different proteins in a time resolved manner. In this field the oSED method, where enzymatic digestion of adsorbed proteins are performed without any preceding desorption procedures [14,15], can prove to be very informative when combined with a carefully designed experimental method.

### vCSF

In this study, we have used vCSF for the development of an *in vitro* adsorption assay. This is a good model system that gives a close-to-real *in situ* adsorption profile similar to applications of *in vivo* sampling with microdialysis or with an implanted biomaterial surface in contact with protein based body fluids. The model system contain: physiological values of pH, ion strength, total protein concentration and naturally occurring interfering substances [12], as well as a surface that is hydrophobic and has a surface roughness encountered in real medical applications. Compared with a model system based on an artificially created standard solution, a vCSF system adds complexity in terms of post-translation modifications such as phosphate groups, oligosaccharides and lipids. The vCSF may also contain proteins that originate from, for example, blood and skin transferred into the solution during the sampling process. All these parameters will influence the adsorption, and can be measured and analyzed by the described method.

The vCSF was analyzed prior to the adsorption study in order to characterize the model system and test the performance of the LC-MS system. In total, 166 proteins were identified (Supplementary Table 1). Compared with a list of the top 41 proteins quantified in CSF by Thompson *et al*. [22], 30 were identified in vCSF. In a further comparison of the ten most abundant proteins identified by Thompson *et al*. [22] in CSF, our results based on vCSF agreed with MS data from a recent study of CSF [16]. A similar pattern between the two studies, in terms of the number of detected peptides for each protein relative to the protein concentration (Table 1) was observed. This certifies that a reasonable prediction is that the most common proteins in CSF also are among the most common in our model system of vCSF. Further, the large dynamic range of protein concentrations in CSF [22] is also applicable for vCSF [23] and must be taken into considerations during adsorption studies.

### Time-dependent adsorption

The potential of the oSED screening method for protein adsorption was demonstrated using PC membranes incubated in vCSF. Membranes were collected after different times of adsorption as schematically described in Figure 1. The oSED method used two experimental replicates for digestion and when combined with MS detection, adsorption profiles of all detectable proteins were achieved for the experimental time series. In addition, it was possible to evaluate a number of characteristics of the studied proteins.

In total, 370 unique proteins were detected. This higher number of identified proteins after adsorption compared with native vCSF solutions is an effect of reduced concentration range in adsorbed samples, due to adsorption of similar amounts of all proteins. Since pure vCSF has orders of magnitudes in difference between the most and least abundant proteins; this effect will increase the number of proteins that can be detected with LC-MS/MS. This observation agrees with a previous study where a higher number of proteins was identified as adsorbed onto the surface compared with unprocessed CSF [14]. The phenomenon also mimics the principle used for the ProteoMiner [24] sample preparation kit, where a library of combinatorial peptide ligands aim to bind similar amounts of all proteins present in a sample. As expected, the number of adsorbed proteins increased with longer incubation time according to Figure 2. After 15 min, 168 proteins (45% of all identified proteins) were identified and after 480 min, the number of identified proteins had increased to 284 proteins (75% of all identified proteins). Hirsh [25] found an ‘overshooting’ tendency of protein adsorption after 40 min in studies of the ‘Vroman effect’ [26] in complex protein solutions. Applying this theory to our data, might explain the curve behavior in Figure 2 at t = 60 min. This effect was, however, not statistically significant. Some fluctuations of the total number of identified proteins can also be an effect of that many proteins were present at threshold concentrations for possible detection and their appearance in MS analysis was therefore sporadic. Such variations are generally observed for MS data and are therefore expected [27].

An alternative type of data evaluation is a 2D graphic representation in which different values are represented by different colors, also known as heat map (Figure 3). In Figure 3, a protein identified at a certain point of time (with the requirement of minimum two unique peptides) was also regarded as identified if only one unique peptide was detected at other time points. This assumption improved the grouping. Proteins were sorted into groups of different adsorption behavior, where group A includes proteins that adsorbed to the surface at the first measuring point (after 15 min of incubation) and thereafter were identified at all other time points. The B group is proteins that adsorbed to the surface after some additional time and thereafter were identified at all times. Group B was further divided into four sub-groups; B_1_ (30–480 min), B_2_ (60–480 min), B_3_ (120–480 min), B_4_ (240–480 min). Group C are proteins that were initially identified but not present at later times. These proteins showed a desorption trend. Group D represents proteins that gave an unclear time-dependent adsorption. A more specified presentation of the identified proteins that had adsorbed at a specific time points and at what level of unique peptides, is given in Supplementary Table 2. Most proteins that adsorbed to the surface at the earliest time point tended to stick to the surface and did not desorb upon longer incubation times. Out of 370 identified adsorbed proteins at any time, 315 (84%) showed a strictly adsorbed-and-stay behavior (Group A and B). All of the ten most abundant proteins in CSF (except α-1-acid glycoprotein 2) had this behavior (Table 1). In addition to the previously mentioned adsorption behavior groups, more than 20 proteins almost followed the adsorbed-and-stay behavior, but were not detected at some time point due to being a low abundant protein in the vCSF, thereby ending up in Group D. These results show that the described method enables screening for differences in the adsorption behaviors among different proteins.

Besides the number of identified proteins, there are different MS data parameters available for evaluating the adsorption. The least manipulated parameters and the easiest to interpret are peptide spectrum match (PSM) and protein sequence coverage, respectively. Additional parameters are, for example, precursor ion area measurements (area under curve) [28] and protein scoring. The latter are linked to peptide charge and number of unique peptides for the studied protein and is directly reported by the search program. In order to be able to record reliable values for the discussed parameters above, it is necessary to use sufficient chromatographic resolution in LC, accurate mass determination in MS and high scan rates [27] to reduce eventual undersampling effects [29]. In this study, we applied a 90 min LC gradient to ensure separation and used high MS resolution (>50,000) to produce reliable identification data while still having a sufficient sampling speed.

To evaluate which MS data to use for illustrating the change in protein adsorption behavior between different proteins, the linear regression of PSM, protein sequence coverage, and protein score versus time was calculated for all proteins in groups A and B (individual protein data not shown). Figure 4A is an illustration of such plot of the sequence coverage versus time for albumin and cystatin-C, two proteins with different size and sequence coverage, but with similar and typical behavior of proteins belonging to group A and B. To further compare how the evaluated parameters for groups A and B change with time, the average slope of the regression trend lines were visualized for each group, see Figure 4B. As seen in Figure 4B, all parameters have similar behavior but with different values. The chosen parameter to represent protein abundance changes was sequence coverage, even though it is not generally recognized as a quantitative measurement compared with PSM. In this case, sequence coverage is, however, slightly advantageous due to the reduction in overestimations from LC-MS measurements of peptides originating from high abundance proteins [30,31]. Further, this parameter is easier to interpret for a broad audience with no specific knowledge in MS, which is an important aim for this paper. The protein sequence coverage for the majority (number of total) of the proteins increased with increased incubation time (data not shown), as exemplified in Figure 4A. However, there are significant (p < 0.05) differences in the slope of the sequence coverage versus time between group A and the following slopes B_1_, B_2_ and B_4_. The protein group B_3_ does not follow the general pattern, probably due to only six proteins identified and to a probable false detection [32]. The similar pattern of protein sequence coverage was also observed for protein score (Proteome Discoverer, Thermo Scientific) and PSM, with even lower p-values (p < 0.01) (Figure 4B).

Molecular weight (kDa) and isoelectric point (pI) are known parameters affecting the adsorption of individual proteins, readily available for comparison from the Proteome Discoverer software. The molecular weight of all 370 adsorbed proteins ranged between 7.8 and 3813 kDa with median size 51.6 kDa. The pI values of all proteins were between 4.41 and 11.65, with median 6.54. This information was also coupled to adsorption behavior for group (A, B_1_, B_2_, B_3_ and B_4_) and as presented in Figure 5, the overall difference in size and calculated pI of the adsorbed proteins did not vary significantly for the different groups and can, in this case, not explain the observed adsorption trends.

#### Limitations of the method

The oSED method is a simple approach, easy to apply to adsorption studies and can act as a powerful complement to existing surface analysis techniques, but it has some limitations. Its current performance does not take into consideration proteins that are still adsorbed after enzymatic digestion. Furthermore, differences in peptide formation between samples due to different trypsin-to-protein substrate ratios [33,34] are difficult to adjust since there is no *a priori* knowledge of the amounts of the adsorbed proteins. These drawbacks do not influence the data exemplifying the method in this paper, but might be an issue for further aspects of quantitative measurements. One important aspect with the use of a complex biological sample is the difficultness to evaluate the oSED method in mechanistic detail. On the other hand, more or less mismatched solutions (compared with vCSF) will induce uncertainties regarding the origin of adsorption/desorption diving force. These areas are of general interest, however, and should be further investigated.

## Conclusion

The presented method is an easy and functional surface-nondestructive tool to qualitatively investigate protein adsorption on basically any type of surface exposed to complex protein solutions.

The established method for studying adsorption in complex protein solution was proved for vCSF studies. The method relates MS data from 370 identified adsorbed proteins to time-dependent adsorption behavior in a way that is both fast and easy to interpret. For example, the number of identified proteins after different time of adsorption as well as the interpretation on how the adsorption of specific proteins changes with time is enabled.

The method was capable to present profiles of the adsorption behavior for all proteins with time. In general, the overall adsorption was concluded to increase with time in the complex protein solutions. An observation specific for the studied adsorption system was the lack of correlation between adsorption and the molecular weight and pI value of the proteins.

## Future perspective

We believe that this use of the presented method can be an important tool for understanding general protein adsorption mechanisms originating from complex mixtures. The aim is to develop the method to be also used for absolute quantification of the adsorbing proteins. This is currently under investigation and is hopefully presented in the near future. In an even broader view, this application of the oSED method may be a significant part of the toolbox for scientists that characterize surfaces in order to screen for particularly low (or high) affinity for certain proteins.

**Table 1.** **Abundant cerebrospinal fluid and ventricular cerebrospinal fluid protein comparison.**

**Roster number**	**Protein**	**mg/l [22]**	**% in CSF [22]**	**Number of peptides**		**Adsorption time (min)**		**Slope^†^**
				**CSF [16]**	**vCSF**	**15**	**480**	
1	Serum albumin	200	67	59	117	21	42	55
2	Prostaglandin-H2 D-isomerase	26	9	7	6	2	6	15
3	Ig γ-1 chain C region	22	7	5	7	3	6	13
3	Ig γ-2 chain C region	22	7	2	8	2	5	17
4	Transthyretin	17	6	2	2	7	10	27
5	Serotransferrin	14	5	35	62	1	3	2
6	α-1-antitrypsin	8	3	10	5	3	11	43
7	Apolipoprotein E	8	3	13	18	10	18	38
8	Apolipoprotein A-IV	6	2	5	3	6	20	61
9	Cystatin-C	6	2	12	18	5	8	48
10	α-1-acid glycoprotein 2	3.6	1	3	0	0	1	0

The number of detected peptides from ten of the most abundant [22] proteins in CSF for a recent MS study [16] and the raw vCSF used as model system for adsorption in this study. The amount of protein specific peptides detected for the first and last adsorption time is presented. The slope of the protein sequence coverage over time for all measuring points (linear regression) for the protein is presented in the table.

^†^The slope is the linear regression of all time points × 10^-3^.

CSF: Cerebrospinal fluid; vCSF: Ventricular cerebrospinal fluid.

Data taken from [22].

**Executive summary**

The presented method is a surface-nondestructive tool, to qualitatively investigate time-dependent protein adsorption on basically any type of surface exposed to complex protein solutions.
The adsorption in complex protein solution is proved for ventricular cerebrospinal fluid, which also was characterized with MS.
The method relates MS data from 370 identified adsorbed proteins to time-dependent adsorption behavior in a way that is both fast and easy to interpret.
The method is capable to present profiles of the adsorption behavior for all identified proteins.
In general, the overall adsorption is concluded to increase with time in the complex protein solutions.
No correlation was observed between the protein adsorption to pI and molecular weight, respectively.
