# Supplementary material for: MS for investigation of time-dependent protein adsorption on surfaces in complex biological samples
Source: Future Sci OA. 2015 Nov 1;1(4):FSO32. doi: 10.4155/fso.15.32 (PMC5137957; doi:10.4155/fso.15.32)
Supplement: Supplementary file 1 [file fso-01-32-s1.docx]

# Supplementary Tables

*Table 1*

vCSF protein abundance list. The 43 most abundant proteins discovered in the vCSF used as protein solution. The ranking is based on the mean% protein sequence coverage of each protein (high to low). The mean numbers of detected unique peptides and mean PSM are also shown for each protein.

| Protein | Sequence Coverage | # Unique Peptide | # PSM |
| --- | --- | --- | --- |
| Serum albumin | 93.3 | 107.4 | 675 |
| Cystatin-C | 72.6 | 17.2 | 72 |
| Ribonuclease pancreatic | 68.1 | 9.6 | 44 |
| Superoxide dismutase | 65.7 | 5.8 | 9 |
| Serotransferrin | 61.5 | 58.8 | 168 |
| Insulin-like growth factor-binding protein 7 | 59.7 | 19.4 | 45 |
| Ig alpha-1 chain C region | 57.8 | 9.4 | 50 |
| Serum amyloid A-1 protein | 53.1 | 4.0 | 6 |
| Kallikrein-6 | 48.0 | 11.0 | 24 |
| ProSAAS | 46.8 | 8.4 | 15 |
| Apolipoprotein E | 46.6 | 15.6 | 22 |
| Hemoglobin subunit beta | 45.6 | 5.0 | 16 |
| Haptoglobin | 44.6 | 17.0 | 35 |
| Vitamin D-binding protein | 43.8 | 23.4 | 40 |
| Ig lambda-3 chain C regions | 40.6 | 5.8 | 8 |
| Beta-2-microglobulin | 39.7 | 6.2 | 15 |
| Osteopontin | 38.7 | 10.6 | 84 |
| Alpha-1B-glycoprotein | 38.5 | 14.0 | 22 |
| Ig kappa chain V-III region WOL | 38.5 | 3.5 | 5 |
| Beta-2-glycoprotein 1 | 38.1 | 12.6 | 37 |
| Ig kappa chain C region | 38.1 | 4.2 | 8 |
| Apolipoprotein A-II | 37.0 | 3.2 | 5 |
| Dickkopf-related protein 3 | 35.2 | 9.0 | 19 |
| Fibrinogen alpha chain | 34.9 | 23.0 | 41 |
| EGF-containing fibulin-like protein 1 | 34.2 | 13.8 | 28 |
| Prostaglandin-H2 D-isomerase | 33.7 | 5.2 | 9 |
| Hemoglobin subunit alpha | 32.8 | 5.0 | 11 |
| Fibulin-1 | 32.7 | 24.6 | 50 |
| Insulin-like growth factor-binding protein 2 | 32.2 | 6.8 | 15 |
| Transthyretin | 32.0 | 2.0 | 4 |
| Clusterin | 31.0 | 23.2 | 55 |
| SPARC | 29.3 | 7.8 | 14 |
| Ig gamma-2 chain C region | 28.7 | 3.2 | 15 |
| Apolipoprotein D | 28.6 | 6.0 | 12 |
| Retinol-binding protein 4 | 27.9 | 6.4 | 16 |
| Complement factor B | 27.9 | 15.0 | 27 |
| Peptidyl-prolyl cis-trans isomerase A | 26.7 | 3.2 | 4 |
| Ig gamma-3 chain C region | 26.0 | 2.0 | 16 |
| Retinoic acid receptor responder protein 2 | 25.8 | 3.6 | 5 |
| Ectonucleotide phosphodiesterase family member 2 | 25.7 | 23.6 | 36 |
| Ig kappa chain V-III region SIE | 24.8 | 2.0 | 2 |
| Hemopexin | 24.5 | 7.4 | 20 |
| Lysozyme C | 24.5 | 3.2 | 4 |

*Table 2*

Heat map of identified adsorbed proteins. A time resolved map of all identified proteins that adsorbs to the surface providing deeper information about the adsorption behavior of the individual proteins compared with the more schematic Fig. 3. The three colors in the heat map indicate zero (white), one (light green) or two or more (dark green) unique peptides identified for a particular protein.
